# Rhinosinusitis in morbidity registrations in Dutch General Practice: a retro-spective case-control study

**DOI:** 10.1186/s12875-015-0332-8

**Published:** 2015-09-11

**Authors:** Ruth Hoffmans, Tjard Schermer, Karin van der Linde, Hans Bor, Kees van Boven, Chris van Weel, Wytske Fokkens

**Affiliations:** Department of Otorhinolaryngology, Academic Medical Center, Amsterdam, The Netherlands; Department of Primary and Community Care, Radboudumc, Nijmegen, The Netherlands; Australian Primary Health Care Research Institute, Australian National University, Canberra, Australia

## Abstract

**Background:**

There is only limited accurate data on the epidemiology of rhinosinusitis in primary care.

This study was conducted to assess the incidence of acute and chronic rhinosinusitis by analysing data from two Dutch general practice registration projects. Several patient characteristics and diseases are related to the diagnosis rhinosinusitis.

**Methods:**

The Continuous Morbidity Registration (CMR) and the Transitionproject (TP) are used to analyse the data on rhinosinusitis in primary practice. Both registries use codes to register diagnoses.

**Results:**

In the CMR 3244 patients are registered with rhinosinusitis and in the TP 5424 CMR: The absolute incidence of (acute) rhinosinusitis is 5191 (18.8 per 1000 patient years). Regarding an odds ratio of 5.58, having nasal polyps is strongest related to rhinosinusitis compared to the other evaluated comorbidities. A separate code for chronic rhinosinusitis exists, but is not in use.

TP: Acute and chronic rhinosinusitis are coded as one diagnosis. The incidence of rhinosinusitis is 5574 or 28.7 per 1000 patient years. Patients who visit their general practitioner with “symptoms/complaints of sinus”, allergic rhinitis and “other diseases of the respiratory system” have the highest chances to be diagnosed with rhinosinusitis. Medication is prescribed in 90.6 % of the cases.

**Conclusions:**

Rhinosinusitis is a common diagnosis in primary practice. In the used registries no difference could be made between acute and chronic rhinosinusitis, but they give insight in comorbidity and interventions taken by the GP in case of rhinosinusitis.

## Background

Rhinosinusitis is one of the commonest reasons for general practice visits and can have a substantial influence on a person’s quality of life [1–4]. Despite the high prevalence and significant morbidity of rhinosinusitis, there is only limited accurate data on the epidemiology of this condition. This is mainly due to the lack of an generally accepted definition for rhinosinusitis and the different patient selection criteria in epidemiological studies.

A taskforce endorsed by the European Academy of Allergology and Clinical Immonology and the European Rhinologic Society has come up with clear unambigious definitons of rhinosinusitis which can be used for epidemiological and clinical research (The European Position Paper of Rhinosinusitis and Nasal Polyps, EPOS) [5, 6]. EPOS is the first combined guideline for primary and secondary medical care [5–7]. The EPOS definition of rhinosinusitis is defined as two or more symptoms one of which should be either nasal obstruction or nasal discharge. Other possible symptoms are facial pain/pressure or impairment of smell. In acute rhinosinusitis (ARS), this condition is present for less than 12 weeks, in chronic rhinosinusitis (CRS) for more than 12 weeks. Recurrent rhinosinusitis is defined as at least 4 episodes of rhinosinusitis within one year with complete resolution of symptoms between the episodes [5, 6].

In Europe, CRS is an underestimated disease. Data on the prevalence of rhinosinusitis in European populations are rare. For this reason the European Union has funded a large epidemiological survey in more than 20 countries, the Global Allergy and Asthma European Network (GA2LEN) survey, which provides the first European epidemiological data on the prevalence of rhinosinusitis. According to this publication, the overall prevalence of CRS by EPOS criteria was 10.9 % [8]. In Portugal a study was done with cadaver specimens with a mean age of death of 77 years. The prevalence of nasal polyps was 5.5 % [9].

General practitioners (GPs) play a vital role in the Dutch health care system. They are the gate-keepers to specialist care. Nearly all inhabitants are registered with a general practitioner. As most of the health problems presented to GPs are not seen by specialists, general practices are important sources of information about common diseases [10]. In a survey by the Netherlands Central Bureau for Statistics 60 per 1000 Dutch inhabitants in 1992 considered themselves to suffer/have suffered from rhinosinusitis [11].

The estimated incidence of ARS in Dutch general practices in 2003 was 16.4 per 1000 men and 33.3 per 1000 women. This means that at total of 131,800 men and 273,000 women were diagnosed with ARS in 2003 [12]. In the “Second National Study”, a report on diseases and interventions in general practice, an incidence of 22.1 per 1000 patients was reported. (15.2 per 1000 men and 28.8 per 1000 women) [13]. In the UK figures of 25 per 1000 patient years have been reported [11]. No differentiation was made between ARS and CRS in these last two reports.

In the current study, two Dutch general practice morbidity registrations projects were used; the Nijmegen Continuous Morbidity Registration (CMR) and the Transitionproject (TP). The aim of our study was to assess the incidence of ARS and CRS diagnosed by GPs by analysing data from these two Dutch general practice registration projects. We also looked at patient characteristics, comorbidity, reasons for consulting the GP and interventions taken by the GP.

## Methods

This retrospective case-control study did not need approval of an ethical board since the anonymous participants in the already existing database were not submitted to investigations or actions as part of this study.

## General practice morbidity registrations

We used the databases of the following two general practice morbidity registrations to estimate the incidence of ARS and CRS. Permission was granted to access both databases.

### Nijmegen Continuous Morbidity Registration

The CMR involves four general practices in the region of Nijmegen in the Netherlands. The goal of the CMR is to generate epidemiological numbers concerning diseases in the general practice population for the purpose of education and scientific research. Since 1971 all common diseases and all referrals to specialists are entered in this registration, as are all hospital admissions [14]. Background information like date of birth, gender, socioeconomic status, date of practice entry, date and reason of leaving the practice is also registered. Socioeconomic status is divided in three social classes, which are based on the occupation of the wage earner (based on a classification of the Institute for Applied Sociology).

**Fig. 1:**
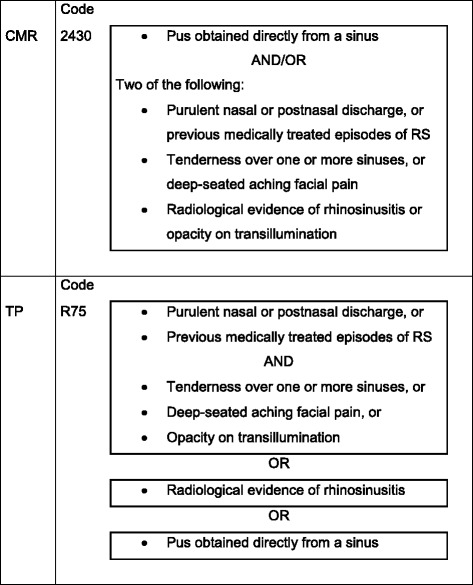
Inclusion in rubric rhinosinusitis

For the current study we used CMR data from 1985 until 2006 comprising an average population of approximately 12,000 patients and 275,602 patient years. All patients who had been diagnosed with rhinosinusitis were included in this study. In the CMR a list of codes based on the E-list (compatible with the ICHPPC-2-defined criteria (ICHPPC: International Classification of Health Problems in Primary Care)) is used (Fig. 1). In the CMR, separate codes for ARS and CRS exist. However, the code for CRS is not used consistently (as a result of an agreement between the participating GPs). To indicate whether a visit was for a new episode or for an already existing episode, the GPs in the CMR practices use a special code linked to the diagnosis. When the code for an already existing episode of rhinosinusitis was used, that (same) episode was not included again for calculation of the incidence of rhinosinusitis [14, 15].

### Transition project

The TP’s goal is to develop and apply episode-oriented epidemiology in general practice by coding all diagnoses by the International Classification of primary Care (ICPC). Participating GPs register all contacts between patient and GP and all actions that result from the contact. The data from 1985 until 2002 are based on a population from three practices in the city of Amstelveen and two practices in the province of Friesland with approximately 18,000 patients and 201,137 patient years of observation. Variables that are documented in the TP are patient characteristics, reasons for encounter, interventions initiated by the GP and referrals [14]. Only the kind of intervention was coded, for example prescription of medication, but not exactly which medicament was prescribed. Figure 1 shows the criteria for inclusion in the rubric rhinosinusitis [16].

The code for reason for encounter could represent a complaint or the diagnosis itself. In the latter case, the patient had the suspicion of having that particular disease and reported this to the GP.

In the TP no difference is made between ARS and CRS. However, the length of the episodes of care is registered in the TP and we tried to use this information to discriminate between ARS and CRS.

### Comorbidity

The commonest comorbidities or predisposing conditions for rhinosinusitis mentioned in literature [1, 5, 6, 17] are: viral infections (upper respiratory tract infections), allergic rhinitis, anatomical variations of the nose, immunocompromised state, nasal polyps, asthma/COPD (chronic obstructive pulmonary disease) and dental infections. Nasal polyps may be part of the diagnosis (chronic) rhinosinusitis, but a separate code in which nasal polyps are mentioned exists in both morbidity registrations. These characteristics were included in the study and related to the diagnosis rhinosinusitis.

### Statistical analysis

We analysed the data from the CMR and TP by calculation of odds ratios (odds of comorbidity in rhinosinusitis population/odds of comorbidity in population without rhinosinusitis). Statistically an odds ratio above 1.0 and a 95 % confidence interval not including 0 is a significant association, but maybe not clinically relevant, therefore we considered an odds ratio of more than 3.0 in combination with the lower limit of the 95 % confidence interval above 2 · 0 to be a relevant association.

## Results

### Incidence of ARS and CRS in the CMR

Based on the above mentioned criteria, a total of 3244 patients were found to be registered with one ore more episodes of ARS in the CMR in the period 1985 to 2006.The incidence of ARS in the CMR was 5191, corresponding with 18.8 per 1000 patientyears. ARS incidence varied slightly over the years, with an apparent trend to lower incidence in the period 1989 to 2004. The code for incident cases of CRS was only used in 33 cases (0.1 per 1000 patientyears). The prevalence of ARS and CRS was 5197 (18.9 per 1000 patientyears) and 65 (0.2 per 1000 patientyears) respectively.

The population with rhinosinusitis in the CMR was mainly from the lowest social class (40 · 1 %); 14 · 4 % was from the highest social class.

**Fig. 2:**
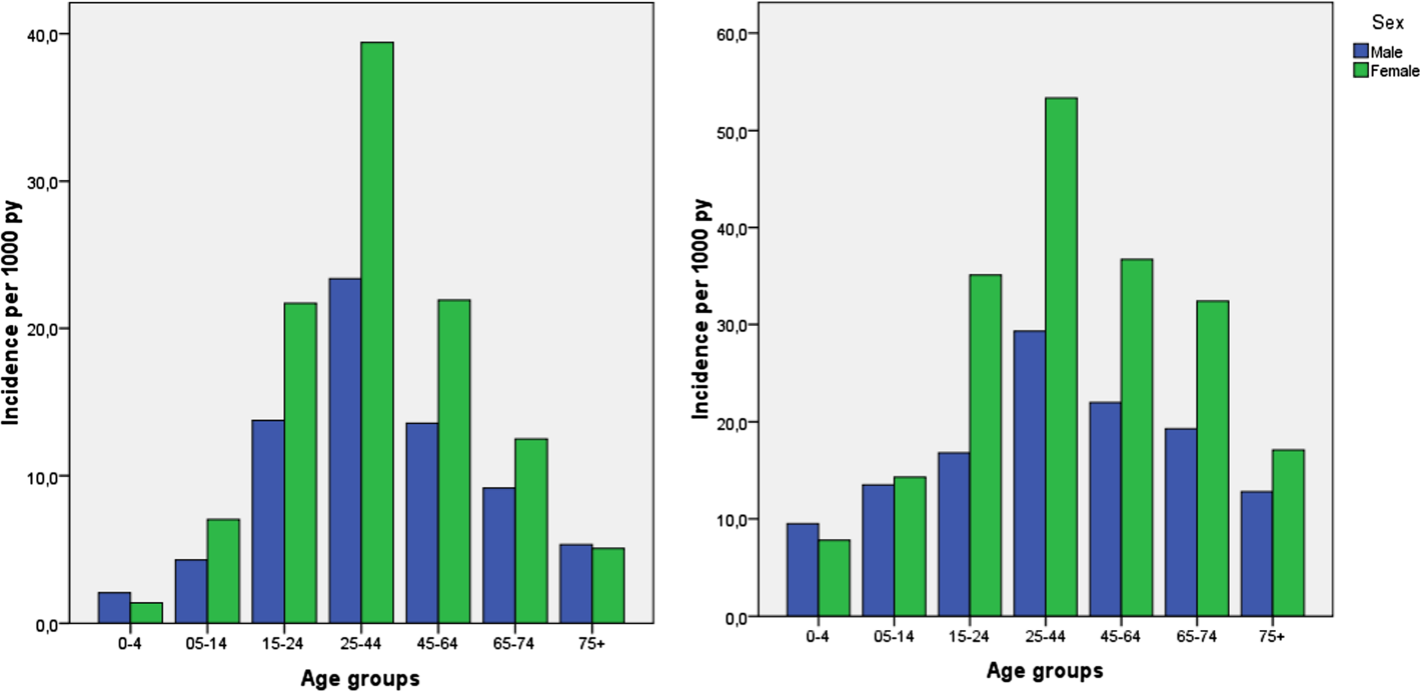
Incidence of acute rhinosinusitis in men and women of different age groups. CMR and TP

The incidence of ARS was unequally distributed over the age groups and sexes. The incidence in men was 14 · 4, in women 23 · 1 per 1000 patient years (Fig. 2). The incidence was highest in the 25–44 years age group, with 39 · 4 per 1000 patient years for women and 23 · 4 per 1000 patient years for men. There were 27 children below the age of 4 who had been diagnosed with ARS.

### Incidence of ARS and CRS in the TP

Reliable determination of the length of episodes was not possible in the TP data, despite the code for the end of an episode. Because an episode can end in between two visits to the GP, the exact end of an episode remains unknown. Therefore no discrimination between ARS and CRS could be made. In the TP 5424 patients had been diagnosed with one or more episodes of rhinosinusitis in the period 1985 to 2002. The total incidence of rhinosinusitis in the TP was 5574, or 28 · 7 per 1000 patient years. The distribution of rhinosinusitis over the age groups and sexes was comparable to the distribution in the CMR (Fig. 2). The incidence in men was 21 · 3 per 1000 patient years, in women 35 · 6 per 1000 patient years. Again, the incidence was highest in the 25–44 years age group. The incidence of rhinosinusitis in women was 53 · 3 per 1000 patient years and in men 29 · 3 per 1000 patient years. In the TP 100 children aged 0–4 years had been diagnosed with rhinosinusitis.

### Number of episodes with rhinosinusitis

GPs of the CMR reported a total of 3244 patients having 5191 episodes of acute rhinosinusitis between 1985 and 2006. Most of these patients (69 %) only had one episode during the period of registration, the rest had one or more relapses. Most of them (18 %) had one documented relapse, one patient even had up to 22 relapses. Only four patients met the criteria for recurrent rhinosinusitis mentioned before.

In the TP database 5424 patients experienced 5774 incident cases of rhinosinusitis.

### Comorbidity and rhinosinusitis

To assess whether comorbidity was related to the incidence of ARS in the CMR database, a few diagnoses were related to the diagnosis “ARS”. Table 1 compares the incidence of comorbidity in the rhinosinusitis group with the incidence of morbidity in the population without rhinosinusitis. The rhinosinusitis population represented 50,888 patient years. With an odds ratio of 5.58 and the lower limit of the 95 % confidence interval being 4.46, “nasal polyps” was the only comorbid condition that was significantly associated with rhinosinusitis. With an odds ratio of 2.88 (95%CI 2.70 to 3.07), allergic rhinitis showed a tendency towards a significant association. Analysis of comorbidity from the TP also showed an association between allergic rhinitis and rhinosinusitis and between “other diseases of the respiratory system” and rhinosinusitis (Table 2). The other selected diseases did not meet the cut-off to confirm a significant association.

**Table 1:** Odds ratio of morbidity for patients with rhinosinusitis relative to controls without rhinosinusitis, CMR

Comorbidity	Odds ratio	95 % CI
Viral infection (without fever)	1.57	1.53–1.62
Allergic rhinitis	2.88	2.70–3.07
Dental infections	1.40	1.29–1.52
Asthma	1.46	1.38–1.54
Nasal polyps	5.58	4.46–6.97

**Table 2:** Odds ratio morbidity (odds morbidity in rhinosinusitis/odds morbidity non-rhinosinusitis), TP

Comorbidity	Odds ratio	95 % CI
Allergic rhinitis	4.06	3.62–4.55
Other disease resp. system (including nasal polyps)	3.63	2.81–4.70
Asthma	2.30	2.03–2.61
Upper resp. tract infections	2.16	1.99–2.34
Emphysema/COPD	1.11	0.84–1.46
Disease of teeth/gums	1.07	0.72–1.58

### Reason for encounter in rhinosinusitis

Analysis of the TP database showed that the commonest reason for encounter before the GP recorded rhinosinusitis as his/her diagnosis was “Symptoms/complaints sinus, including pain”. Other frequent reasons for encounter were upper respiratory tract infections and headache. Children aged 0 to 4 years consulted with a cough or fever relatively often. Adolescents’ (age between 15 and 24 years) top-3 reasons for encounter were cough, headache and symptoms/complaints of the sinus. Patients older than 65 usually came with symptoms/complaints of the sinus, but also relatively often with a cough (Table 3).

**Table 3:** Reason for encounter in rhinosinusitis cases (*n* = 5774). Absolute numbers and percentage per age group. Top 10. TP

			Age group						
	Label reason for encounter	Total N (%)	0–4 N (%)	5–14 N (%)	15–24 N (%)	25–44 N (%)	45–64 N (%)	65–74 N (%)	75+ N (%)
1	Symptoms or complaints of sinus (including pain)^a^	2001 (24.5)	7 (4.6)	60 (11.8)	196 (21.8)	1131 (29.7)	417 (22.7)	133 (20.9)	57 (18.3)
2	Upper respiratory infection (common cold)	1000 (12.3)	16 (10.5)	60 (11.8)	126 (14.0)	464 (12.2)	243 (13.3)	54 (8.5)	37 (11.9)
3	Headache^b^	994 (12.2)	6 (3.9)	84 (16.5)	148 (16.4)	457 (12.0)	192 (10.5)	66 (10.4)	41 (13.2)
4	Cough	947 (11.6)	38 (24.8)	100 (19.6)	90 (10.0)	333 (8.7)	211 (11.5)	121 (19.1)	54 (17.4)
5	Sinusitis, acute or chronic^c^	784 (9.6)	3 (2.0)	9 (1.8)	69 (7.7)	429 (11.3)	218 (11.9)	43 (6.8)	13 (4.2)
6	Fever	386 (4.7)	35 (22.9)	60 (11.8)	31 (3.4)	141 (3.7)	70 (3.8)	30 (4.7)	19 (6.1)
7	Symptoms or complaints of throat	237 (2.9)	2 (1.3)	13 (2.5)	43 (4.8)	88 (2.3)	64 (3.5)	20 (3.1)	7 (2.3)
8	Medication/prescription/injection	218 (2.7)	1 (0.7)	4 (0.8)	10 (1.1)	115 (3.0)	71 (3.9)	13 (2.0)	4 (1.3)
9	Sneezing/nasal congestion	211 (2.6)	4 (2.6)	17 (3.3)	36 (4.0)	81 (2.1)	37 (2.0)	26 (4.1)	10 (3.2)
10	General weakness/tiredness	181 (2.2)	8 (5.2)	20 (3.9)	21 (2.3)	73 (1.9)	29 (1.6)	17 (2.7)	13 (4.2)

^a^Label “Symptoms or complaints of sinus”: patients present themselves with complaints

^b^excluded were: N02 = Tension headache. N89 = Migraine. R09 = Sympt/complt sinus

^c^Label “Sinusitis”: patients present themselves with the suspicion of having rhinosinusitis

### GPs’ interventions for rhinosinusitis

Table 5 shows the diagnostic assessment and interventions of the GP for patients with rhinosinusitis from the TP database. GPs medically examined most patients and almost 91 % received a prescription for medication to treat the rhinosinusitis. Unfortunately, no details were recorded about the precise examinations the GPs performed and the medication that was prescribed. Of all patients diagnosed with rhinosinusitis by the GPs, 7 · 6 % was sent for diagnostic radiology.

Young children (aged 0–4 years) received less prescriptions for medication than patients in other age groups, but were referred more often than patients from other age groups (Table 4). Of the total population 2 · 7 % was referred to a medical specialist. A higher percentage of children, aged between 0 and 4 was referred.

**Table 4:** Percentage of rhinosinusitis cases (*n* = 5774) with interventions. TP

		Age group							
	Label	Total	0–4	5–14	15–24	25–44	45–64	65–74	75+
1	Medical examination or health evaluation	91.3	95.0	94.0	94.2	90.5	89.5	93.2	94.0
2	Medication prescription or injection	90.6	84.0	86.3	90.4	90.0	92.4	92.7	92.1
3	Advice or health education	22.3	18.0	26.6	23.5	24.4	17.9	18.1	24.1
4	Diagnostic radiology/imaging	7.6	9.0	9.9	7.1	7.3	8.1	6.8	6.9
5	Referral to medical specialist or hospital	2.7	9.0	2.1	2.0	2.3	3.1	3.3	4.2

## Discussion

### Main Findings

Although clear unambigious definitons of rhinosinusitis have been published, the diagnosis of rhinosinusitis in general practice remains complicated. Firstly the discrimination between rhinosinusitis and other upper airway diseases is difficult [5, 6, 18]. The symptomatology of rhinitis and rhinosinusitis overlap. When the patient has nasal blockage, purulent discharge and/or facial pain, it may be impossible to make an adequate diagnosis without nasal endoscopy or CT scan, none of which are usually available in the GP practice [19, 20]. It was found that questionnaire-based and clinical based CRS show moderate correlation [21]. On the other hand, symptom-based CRS (based on EPOS criteria) has been shown to be significantly associated with positive endoscopy in nonallergic subjects [22].

In the two registries the GPs do not seem to differentiate between ARS and CRS, which may just be a matter of limitations of the studied registries. In a previous study from our group 69 % of Dutch GPs reported to discriminate between ARS and CRS. However, their definitions of ARS and CRS varied [23].

Almost 91 % of the patients with rhinosinusitis received a prescription for medication. Antibiotics are still prescribed quite often for this indication [23], even though we know that antibiotics do not influence the clinical course of sinusitis nor the rate of relapses during 1-year follow-up [24, 25]. Initial management can be limited to symptomatic treatment only [26, 27]. In 7 · 6 % of the rhinosinusitis patients in our study diagnostic radiography was performed. In ARS, X-rays have no prognostic value nor therapeutical consequences [26]. In patients with clinical diagnosis of ARS it has been shown that less than half actually have significant abnormalities at X-ray examination [28].

From the data of the TP, it seems that young children are referred more easily than patients in other age groups. A likely explanation for this observation is that GPs are more cautious when they treat very young children. However, the analysis of a subgroup of only 100 children is not as reliable as the analysis on the other (larger) age groups.

### Strengths and limitations of this study

It is possible that the incidence of rhinosinusitis in this study is overestimated, because the diagnosis is only based on symptoms and physical examination by the GP. For the diagnosis we depend on the GP’s assessment, we are not sure that inclusion criteria are strictly followed. Based on sinus puncture/aspiration (which is considered the most accurate diagnostic test), 49–83 % of a population of symptomatic patients was proven to have ARS [29]. Furthermore, we do not know whether patients who presented with a “second” episode had complete resolution of the symptoms in between their contacts with the GP. Therefore differentiation between “recurrent” ARS and CRS is not possibible. On the other hand, incidence could be underestimated, because many patients with complaints, and possibly rhinosinusitis, do not visit their GP.

Questionnaire-based studies on rhinosinusitis exist showing a prevalence of, for example, CRS of 10.9 % in Europe and even 14.3 % in Amsterdam, the Netherlands [8]. This is much higher than the numbers found in current study for rhinosinusitis overall (ARS and CRS together), but it is known that questionnaire-based and clinical-based CRS show only moderate correlation [21].

Unfortunately, we could not discriminate between ARS and CRS in either of the two registries used. In the CMR, there is a separate code for chronic rhinosinusitis but the GPs from the CMR have decided not to use this code. In the TP it depends on the assessment of the GP whether a visit for an episode of rhinosinusitis following an earlier episode is considered a new episode or part of the same episode. Furthermore, it is not possible to determine the end of an episode, since the patient can recover in a period between contacts with the GP. Therefore it was impossible to determine the duration of rhinosinusitis episodes properly.

The incidence of rhinosinusitis in the TP is higher than the incidence in the CMR. Due to missing values in the TP, further statistical analysis of this difference was not possible. A possible explanation for the difference could be the fact that in the TP, the diagnosis is coded as acute/chronic rhinosinusitis. All diagnoses related to rhinosinusitis fit into this group. In the CMR, there are separate diagnose codes for ARS and CRS. The code for CRS is not used, but certain symptoms/complaints concerning the sinus do not fit into the ARS group and are probably coded otherwise. Furthermore, the criteria for inclusion in the rubric rhinosinusitis were less strict in the TP.

Not all predisposing factors could be analysed, because of their low incidence in the databases. Immunocompromised state, for example, was too uncommon to analyse. Other conditions had no separate code in the registries. Therefore these conditions could not be compared to the data of the TP. In both registries anatomical variations of the nose were not specifically coded and therefore could not be analysed. Another limitation of this study is that our results can not be easily compared to data of other studies, because it appears that this kind of analysis of GP registries has not been done before.

Ideally registries with clear inclusion criteria for rhinosinusitis, using the unambiguous definitons of rhinosinusitis as defined in EPOS, should be used in a study like this. Information on interventions should be more precise, giving more insight in the medicaments prescribed and the diagnostic radiology that is applied for.

### Interpretation of findings in relation to previously published work

Okkes et al. compared data from a general population health survey of the Dutch Central Bureau for Statistics (CBS) about episodes of chronic diseases experienced by the respondents with data from general practice registration projects. The health survey resulted in higher frequencies than the GP registration for respiratory disorders, including rhinosinusitis (mostly in the age group of 25–44 years). In the CBS health survey 60 per 1000 inhabitants in the Netherlands in 1992 self-reported a diagnosis of rhinosinusitis. These numbers were compared to the numbers of three GP registries showing prevalences between 21 and 31 per 1000 patient years [11]. The differences between men and women and age groups found in this study confirm data found in the Second National Study [13]. The reason for the difference between men and women is still unclear [30]. Most of the predisposing factors for rhinosinusitis found in the literature, like nasal polyps, allergic rhinitis and other diseases of the respiratory system, were also predisposing factors in the current study [1, 5, 6, 17].

In the Dutch guideline for rhinosinusitis, GPs are advised to do a medical examination only in case of long-lasting or severe complaints [30]. It is remarkable that 91 · 3 % of the patients with an incident episode was examined by the GP. It is also remarkable to see that 90 · 6 % of these patients got a prescription for medication. Unfortunately, we do not know which medication was prescribed. Decongestants, antibiotics, analgesics, nasal steroids and antihistamines are some of the commonly prescribed treatments, but cannot be confirmed by this study [1, 5, 6, 23, 30]. These numbers are comparable to the result of an observational study on acute maxillary sinusitis in France and Asia [31, 32].

### Implications for future research, policy and practice

The guideline on rhinosinusitis of the Dutch College of General Practitioners did not discriminate between ARS and CRS until Octobre 2014 [30]. A considerable amount of data suggests that ARS and CRS are independent diseases with different treatments [5, 6, 33]. Therefore, a guideline discriminating between ARS and CRS would be better. Since Octobre 2014 a new guideline for GPs has been published in which the word “acute” is added to the title “rhinosinusitis”. Still there is no separate guideline for CRS [34].

To evaluate the management of rhinosinusitis of the GP in more depth, we conducted a study with additional information on e.g. medication policy [23].

## Conclusions

Rhinosinusitis is a common diagnosis in general practice. Based on two morbidity registrations in general practice, the diagnosis can be related to several other diagnoses as allergic rhinitis and nasal polyps. Medication is prescribed in 91 % of the cases and almost 8 % is sent for diagnostic radiology.

Based on the two general practice registries and the Dutch GP guidelines, GPs do not seem to make a difference between ARS and CRS. The incidence of these two diseases could not be assessed separately. Because the different pathophysiology, diagnosis and treatment of these entities, this would deprive patients with rhinosinusitis of optimal care.
